# *Lithocarpus dahuoaiensis* (Fagaceae), a new species from Lam Dong Province, Vietnam

**DOI:** 10.3897/phytokeys.69.9821

**Published:** 2016-08-18

**Authors:** Nguyen Van Ngoc, Luong Van Dung, Shuichiro Tagane, Hoang Thi Binh, Hoang Thanh Son, Vo Quang Trung, Tetsukazu Yahara

**Affiliations:** 1 Center for Asian Conservation Ecology, Kyushu University, 744 Motooka, Fukuoka, 819-0395, Japan; 2 Department of Biology, Dalat University, 01 – Phu Dong Thien Vuong, Dalat, Vietnam; 3 Silviculture Research Institute, Vietnamese Academy of Forest Sciences, Ha Noi, 10999, Vietnam; 4 Dong Nai Culture and Nature Reserve, Ma Da, Vinh Cuu, Dong Nai, Vietnam

**Keywords:** Da Huoai, Fagaceae, Lam Dong Province, Lithocarpus, Lithocarpus dahuoaiensis, Vietnam

## Abstract

*Lithocarpus dahuoaiensis* Ngoc & L. V. Dung, a new species from the Central highland of Vietnam, is described and illustrated. The new species is morphologically similar to *Lithocarpus macphailii* (M. R. Hend.) Barnett or *Lithocarpus encleisocarpus* (Korth.) A. Camus in having completely entire leaf margin, solitary cupule, long stalks of fruits, deeply cup-shaped or turbinate cupules, with a number of horizontal filiform lines. The species differs in its nut enclosure ca. 1/2 – 2/3 of the nut, adaxially glabrous leaf blades, secondary veins 11–12 pairs and faintly to very faintly visible hairs on the outside of the cupule. A table showing the morphological comparison of *Lithocarpus dahuoaiensis* with *Lithocarpus macphailii* and *Lithocarpus encleisocarpus* is also provided.

## Introduction

*Lithocarpus* Blume is the second largest genus of the family Fagaceae, comprising 341 species (The Plant List 2013). The genus is commonly known as Stone Oaks and widely distributed throughout the tropical and sub-tropical broad-leaved evergreen forests in East and Southeast Asia, extending to New Guinea (Cannon 2001, Phengklai 2008). In North America, one species of *Lithocarpus*, *Lithocarpus densiflorus* (Hook. & Arn.) had been known, but has recently been treated as a member of a new monotypic genus *Notholithocarpus* (Manos et al. 2008). The center of diversity is in East to Southeast Asia, where 123 species are enumerated in China (Huang et al. 1999), 58 species in Thailand (Phengklai 2008, Strijk et al. 2014) and 115 species in Vietnam (Ho 2003, Ban 2005).

In Vietnam, the species of Fagaceae are highly diversified and can be seen in various forest types, from dry evergreen forest at lowland to montane evergreen forest at high mountains. A total of 216 species and two varieties in six genera have been recorded in the country (Ho 1999, Ban 2005, Linh et al. 2013, Vuong and Xia 2014), which represents 66% of the total world genera and 24% of the total world species diversity in this family. One species of *Fagus* L., two species of *Castanea* Mill., 54 species of *Castanopsis* (D. Don) Spach., 43 species of *Quercus* L., one species of *Trigonobalanus* Forman and 115 species with two varieties of *Lithocarpus* have been found, indicating that *Lithocarpus* is the largest and most diversified genus of the family in Vietnam. Recently, several taxonomic works on Fagaceae of Vietnam were published (Deng et al. 2010, Linh et al. 2013, Vuong and Xia 2014), indicating that taxonomic studies of the family Fagaceae in Vietnam are still required.

**Figure 1.**
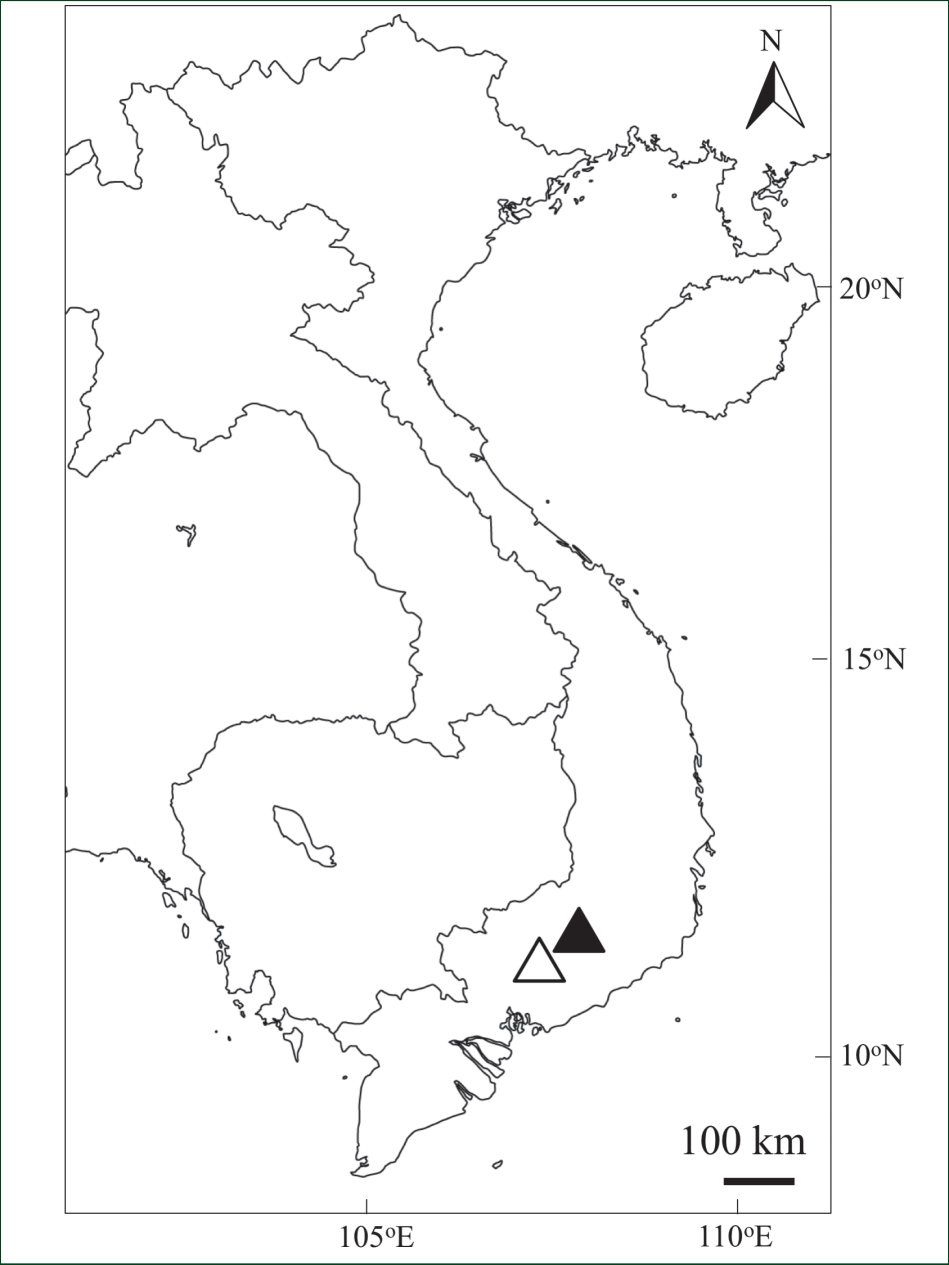
Distribution map of *Lithocarpus dahuoaiensis* Ngoc & L. V. Dung. Black triangle, Da Huoai, Lam Dong Province (Type locality); White triangle, Dong Nai Culture and Nature Reserve, Dong Nai Province.

Lam Dong Province is located in Central highland of Vietnam (Fig. 1) and has long been known as one of the biodiversity hotspots in Vietnam. In June 2015, the International coordinating Council of UNESCO's Man and the Biosphere Program added 20 new sites to the World Network of Biosphere Reserves, among which Langbiang biosphere reserve in Lam Dong Province was one of the sites selected (UNESCO 2015). In the region, 3,490 species of vascular plants have been recorded, including 131 and 45 threatened species which are listed in Vietnam's Red Book and IUCN Red List Categories, respectively (Ban et al. 2007, IUCN 2012). As for Fagaceae, 90 species, including 30 species of *Lithocarpus*, are recorded from Lam Dong Province (Ho 2003, Ban 2005, Dung 2005).

During our floristic inventory in Lam Dong Province in 2015, we discovered several individuals resembling species of the genus *Lithocarpus*. Further study revealed that these did not resemble any species described previously. Here, these are described and illustrated as *Lithocarpus dahuoaiensis* Ngoc & L. V. Dung, sp. nov.

## Material and methods

The new species was discovered through literature review, as well as a thorough examination of specimens in the herbaria at ANDA, BKF, DLU, FU, HN, K, KYO, L, P, VNM and digital images of specimens on JSTOR Global Plants (https://plants.jstor.org/), Herbier National de Paris, Muséum National d'Histoire Naturelle (P).

## Taxonomy

### Lithocarpus dahuoaiensis

Taxon classificationPlantaeFagalesFagaceae

Ngoc & L. V. Dung sp. nov.

urn:lsid:ipni.org:names:60472844-2

Figs 2 , 3

#### Diagnosis.

*Lithocarpus dahuoaiensis* is morphologically similar to *Lithocarpus macphailii* (M.R.Hend.) Barnett and *Lithocarpus encleisocarpus* (Korth.) A. Camus in having a completely entire leaf margin, solitary cupule, long stalks of fruits, deeply cup-shaped or turbinate cupules with the number of horizontal filiform lines. But *Lithocarpus dahuoaiensis* is distinct by its cupules enclosing ca. 1/2–2/3 of the nuts (vs. cupules almost completely covering the nut in *Lithocarpus macphailii* and *Lithocarpus encleisocarpus*), surface of the cupule densely tomentose inside and subtle hairy to very subtle hairy outside (vs. outside densely fulvous tomentose in *Lithocarpus macphailii* and outside densely fulvous tomentose by stellate hairs in *Lithocarpus encleisocarpus*), leaf blades glabrous adaxially, undersides covered with very short soft hairs and subtle (vs. densely glaucous tomentose with adpressed, stellate hairs abaxially in *Lithocarpus macphailii*, pubescent then glabrescent abaxially in *Lithocarpus encleisocarpus*), secondary veins 11–12 pairs (vs. 12–16 pairs in *Lithocarpus macphailii* and 8–10 pairs in *Lithocarpus encleisocarpus*).

#### Type.

VIETNAM. Lam Dong Province, Da Huoai, along the 20 National Highway, in the lowland evergreen forest, alt. 225 m, 11°23'32.5"N, 107°33'56.3"E, 14 June 2015, *N. Nguyen, D. Luong, B. Hoang, T. Nguyen V3194* (holotype: KYO!; isotypes: DLU!, FU!, HN!, K!, P!, VNM!).

#### Description.

Evergreen tree, up to 35 m tall; young branchlets pubescent with white hairs, soon glabrous, greyish green *in vivo* and blackish brown *in sicco*; terminal buds ca. 10–12 mm long, bud scale 4–6 mm long, densely covered with whitish hairs. Stipules not seen. Leaves alternate, blades broadly elliptic to slightly obovate, ca. 15–27 × 6–11 cm, thickly coriaceous, base cuneate, margin entire, slightly recurved, apex acuminate or caudate, acumen ca. 5–10 mm long, glabrous adaxially, subtle short soft hairs abaxially; midrib slightly raised above, distinctly raised below glabrous, greenish yellow *in vivo*, reddish brown *in sicco*; secondary veins 11–12 pairs, clearly visible on both sides, flat to slightly prominent adaxially, prominent abaxially, veins curving smoothly and disappearing near margins, at an angle of 55–65 degree from the midrib, tertiary veins scalariform, invisible to faintly visible on both surfaces; petioles ca. 10–15 mm long, rounded, thickened, pubescent when young, glabrescent later. Flowers not seen. Infructescences erect, woody, 25 cm long, rachis densely adpressed hairy. Acorn solitary, ovoid or turbinate, 13–15 mm in height, 20–23 mm in diam. (including cupule); fruiting stalk 3–5 mm long, densely fulvous tomentose hair. Cupules, turbinate, base a little broader than the upper part, densely tomentose inside and invisible or subtle hairy outside, lamellate, wall woody, sometimes crackled, enclosing ca. 1/2–2/3 of the nut, 12–14 mm in height, 19–22 mm in diam., bractlets triangular, obscure, forming 6–7 dimly concentric flanges. Mature nut 19–22 mm in height, 20–23 mm in diam., densely white tomentose; scar created by cupule at the base is deeply concave, ca. 13–15 mm in diam.; wall woody, crackled; apex abruptly acuminate, ca. 1.5–2 mm in height.

**Figure 2.**
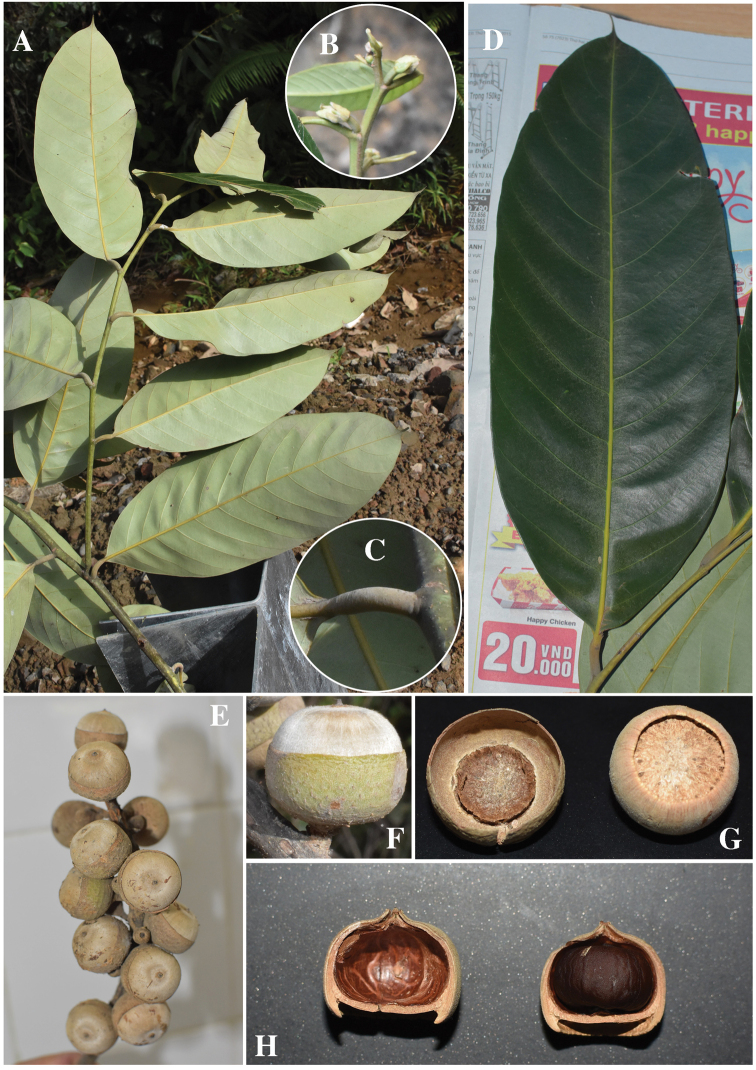
*Lithocarpus dahuoaiensis* Ngoc & L. V. Dung. **A** Leafy twig **B** Buds **C** Petiole **D** Abaxial surface of mature Leaf **E** Infructescence **F** Mature fruit **G** Cupule (left) and bottom of nut (right) **H** Vertical sections of nut.

**Figure 3.**
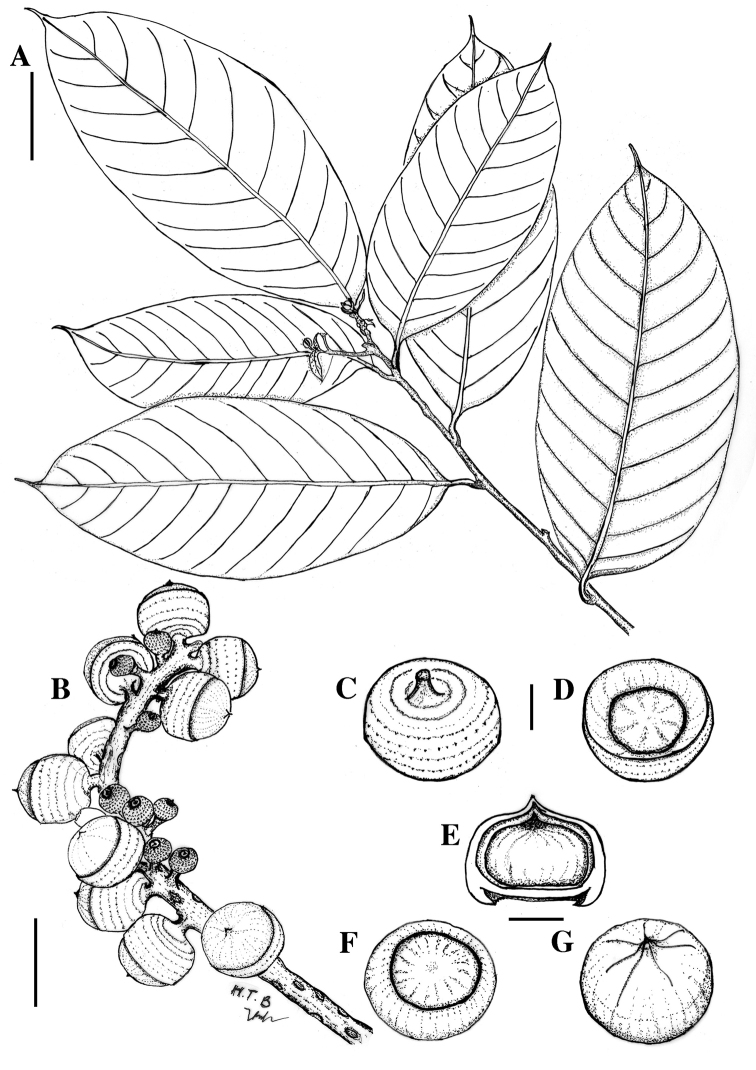
Line drawing of *Lithocarpus dahuoaiensis* Ngoc & L. V. Dung. **A** Leafy twig **B** Infructescence **C**, **D** Cupule **E** Vertical section of mature nut **F**, **G** Mature nut. Scale bars **A**, **B** = 5 cm; **C**–**G** = 10 mm.

#### Phenology.

Mature fruits were collected in June.

#### Distribution and habitat.

Vietnam (so far known from Lam Dong Province and Dong Nai Province split by a boundary along National highway 20). (Figure 1)

#### Etymology.

The specific epithet is derived from the type locality, Da Huoai, Lam Dong Province, Central Highland Vietnam.

#### Conservation status.


 Data Deficient (DD). Three fruiting individuals were found at the type locality, along the Chuoi pass of the 20 National highway. In addition, a staff member of Dong Nai Culture and Nature Reserve has collected this species at Ma Da, Vinh Cuu, Dong Nai Province, indicating its wide distribution around the type locality. However, at present we have no reliable information on its population size. Further investigations are needed to determine the conservation status and actual population size in its natural habitat.

**Table 1.** Morphological comparison between *Lithocarpus dahuoaiensis* Ngoc & L. V. Dung, sp. nov. with *Lithocarpus macphailii* (M.R.Hend.) Barnett and *Lithocarpus encleisocarpus* (Korth.) A.Camus. (The measurements of *Lithocarpus macphailii* and *Lithocarpus encleisocarpus* derive from Soepadmo 1972)

Characters	*Lithocarpus dahuoaiensis*	*Lithocarpus macphailii*	*Lithocarpus encleisocarpus*
Leaf margin	Entire	Entire	Entire
Leaf surface	Glabrous above, very short soft hairs and subtle beneath	Densely glaucous tomentose with adpressed, stellate hair on lower surface	Subglabrous on upper surface, densely glaucous adpressed stellate-hairy on lower surfaces
Leaf size (cm)	15–27 × 6–11	15–22 × 6–8	12–15 × 4–6
Length of petioles	10–15 mm long	10–17 mm long	5–15 mm long
Number of secondary veins	11–12 pairs	12–16 pairs	(7–)8–10(–12) pairs
Length of fruit stalk	3–5 mm long	Up to 5 mm long	10–15 mm long
Acorn size (in diam.)	20–23 mm	20–25 mm	20–27 mm
Cupule size	12–14 mm high × 20–23 mm across	7–15 mm high × 20–30 mm across	N/A
Cupule outside	Faintly or very faintly visible hairs	Densely fulvous-tomentose	Densely fulvous tomentose by stellate hairs
Horizontal rings in cupule	6–7, dimly concentric flanges	5–8, thin, more or less concentric	5–7, more or less concentric
Nut enclosure	Enclosing ca. 1/2– 2/3 of the nut	Almost completely covering the nut	Completely enclosing the nut
Infructescence length	15–25 cm long	10–25 cm long	8–20 cm long

## Supplementary Material

XML Treatment for Lithocarpus dahuoaiensis
